# Cathepsin S are involved in human carotid atherosclerotic disease progression, mainly by mediating phagosomes: bioinformatics and *in vivo* and *vitro* experiments

**DOI:** 10.7717/peerj.12846

**Published:** 2022-02-08

**Authors:** Hailong Wang, Haiying Jiang, Xian Wu Cheng

**Affiliations:** 1 Department of Cardiology and Hypertension, Yanbian University Hospital, Yanji, Jilin, China; 2 Department of Community Health & Geriatrics, Nagoya University Graduate School of Medicine, Nagoya, Japan; 3 Department of Department of Physiology and Pathophysiology, Jiaxing University Medical College, Jiaxing, Zhejiang, China

**Keywords:** Atherosclerosis, Phagosome, Macrophage, Bioinformatics, Cathepsin S

## Abstract

**Background:**

Atherosclerosis emerges as a result of multiple dynamic cell processes including endothelial damage, inflammatory and immune cell infiltration, foam cell formation, plaque rupture, and thrombosis. Animal experiments have indicated that cathepsins (CTSs) mediate the antigen transmission and inflammatory response involved in the atherosclerosis process, but the specific signal pathways and target cells of the CTSs involved in atherosclerosis are unknown.

**Methods:**

We used the GEO query package to download the dataset GSE28829 from the Gene Expression Omnibus (GEO) and filtered the data to check the standardization of the samples through the box chart. We then used the ‘limma’ package to analyze between-group differences and selected the corresponding differentially expressed genes of CTSs from the protein-protein interaction (PPI) network constructed with the STRING database, and then visualized the CTS-target genes. The best matching pathway and target cells were verified by a male mouse ligation experiment, single-sample GSEA (ssGSEA) analysis, and vitro experiment.

**Results:**

There were 275 differentially expressed genes (DEGs) selected from the GSE28829 dataset, and the DEGs were identified mainly in the PPI network; 58 core genes (APOE, CD74, CP, AIF1, *etc*.) target three selected CTS family members (CTSS, CTSB, and CTSC). After the enriched analysis, 15 CTS-target genes were markedly enriched in the phagosome signaling pathway. The mouse experiment results revealed that the percentages and numbers of monocytes and neutrophils and the number of CD68^+^ cells in CTSS deficiency (CatS^−/−^) group were lower than those in the wildtype (CatS^+/+^) group. CTSS mediating phagosome *via* macrophage were further verified by ssGSEA analysis and vitro experiment.

**Conclusions:**

CTSS are the main target molecules in the CTS family that are involved in atherosclerosis. The molecule participate in the progression of atherosclerosis by mediating the phagosome *via* macrophage.

## Introduction

Cathepsins (CTSs) are members of the cysteine protease family in the cytoplasm. Eleven types of CTSs have been identified in humans (Cheng et al., 2011). Sukhova et al. (2003) demonstrated that cathepsin S (CTSS) deficiency can slow the progression of atherosclerosis in a low-density lipoprotein (LDL) receptor-deficiency-induced atherosclerosis mouse model. Other members of the CTS family (*e.g*., CTSL and CTSK) have been shown to be involved in the process of atherosclerosis by mediating inflammation and regulating the immunity response (Lutgens et al., 2006; Kitamoto et al., 2007). CTSs have thus exhibited a strong link to atherosclerosis, but the detailed mechanisms and target cells for CTSs in the progression of atherosclerosis are largely unknown.

It was reported that CTSS activity regulates antigen presentation in atherosclerosis (Borghese & Clanchy, 2011). In addition, CTSB was shown to strongly colocalize with macrophages, a finding which is consistent with those of earlier murine atherosclerosis studies; it also demonstrated that macrophage-derived CTSB plays an important role in atherosclerotic diseases (Jaffer et al., 2008). Another member of the CTS family, CTSD, was reported to play an important role in cholesterol trafficking in atherosclerosis (Liu et al., 2017). Although there is some evidence indicating a relationship between CTS and macrophages and phagosomes, a macro-perspective on the relationship between the entire CTS family and these possible cells and pathways are far from clear.

High-throughput sequencing techniques can clarify the expression of human atherosclerosis-related genes and provide a theoretical reference for screening target genes. A study using the dataset GSE28829 revealed that macrophages do not polarize M1 or M2 but do affect the expressions of the genes MMP9, TLR2, and gp91phox to further accelerate the progression of atherosclerotic plaques (Canton, 2014; da Rocha, De Bastiani & Klamt, 2014; Su et al., 2021). Indeed, our investigation clarified that the expressions of MMP9, TLR2, and gp91phox were high in a murine model of atherosclerosis (Wang et al., 2019). Together these studies showed that GSE28829 is an ideal dataset for studying the pathways and target cells linked to the CTS family in atherosclerosis.

The primary aim of our present study was thus to investigate the expression of CTS-target genes in atherosclerotic plaques, with a special focus on the specific signal pathways and target cells for CTS-target genes.

## Materials and Methods

### Microarray datasets

**Figure 1:**
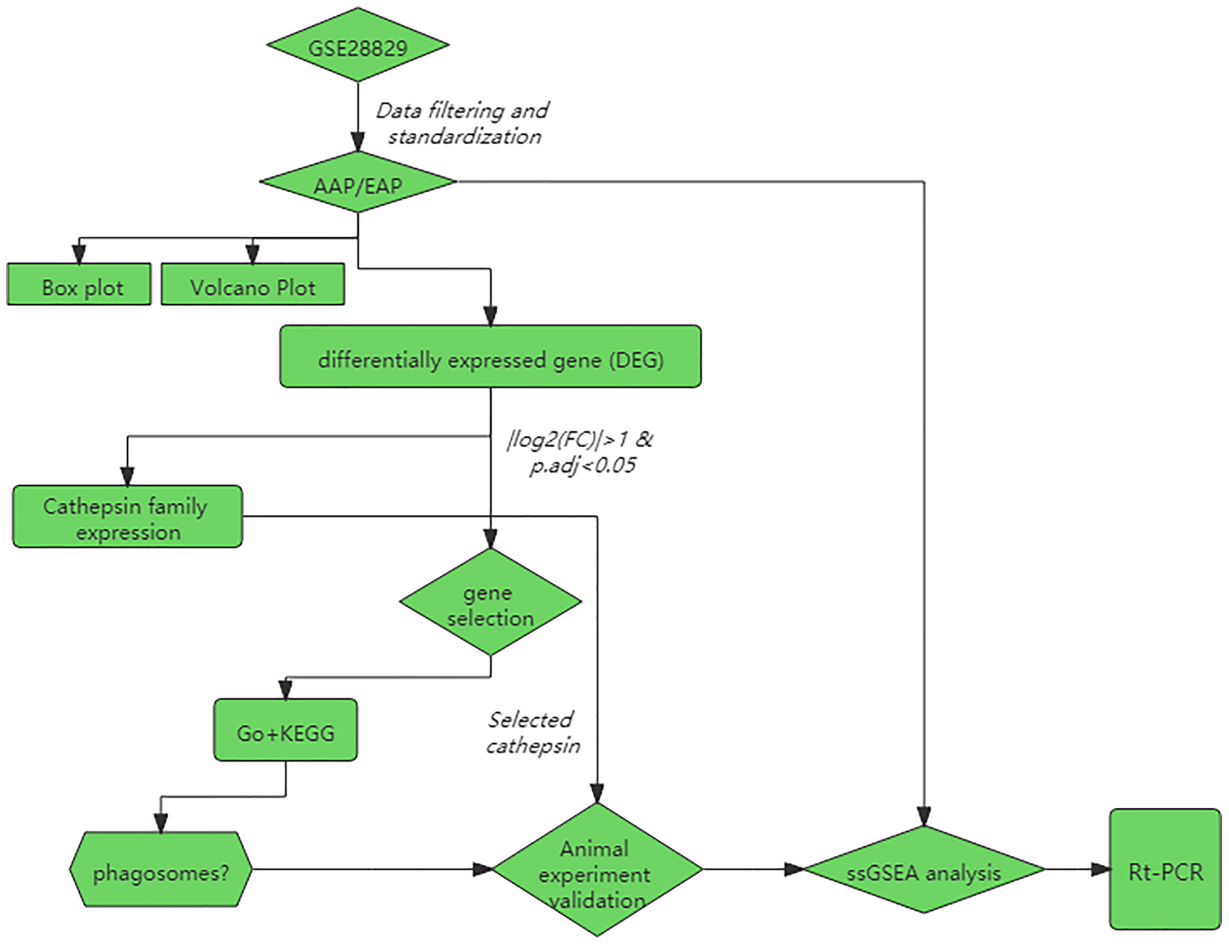
Flowchart of the data analysis and animal experiments. AAP, advanced carotid atherosclerotic plaques; EAP, early carotid atherosclerotic plaques; GO, gene ontology; KEGG, Kyoto Encyclopedia of Genes and Genomes.

We used R software (ver. 3.6.3) to download GSE28829 from the Gene Expression Omnibus (GEO) database (https://www.ncbi.nlm.nih.gov/geo/) through the GEOquery package. The GSE28829 expression profile consists of 16 advanced and 13 early-stage plaques, detected by the Affymetrix Human Genome U133 Plus 2.0 Array. The experimental design is depicted in the form of a flow chart in Fig. 1.

### Data preprocessing and DEG screening

**Figure 2:**
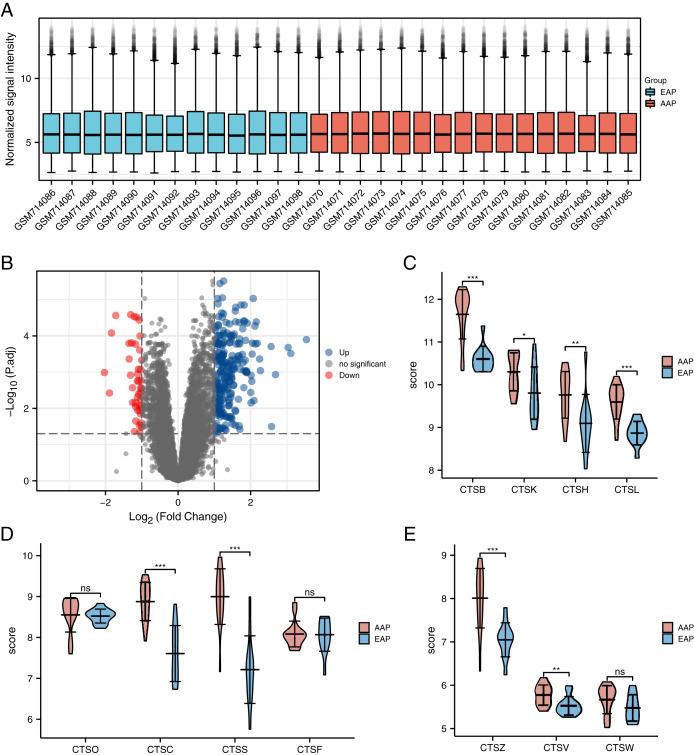
The distribution of the expressions of the cathepsin (CTS) family genes in the GSE28829 dataset. (A) GSE28829 standardized box plot. Each box contains a *black line* representing the median gene expression level, which was at almost the same level across all samples. *Red:* The advanced carotid atherosclerotic plaques (AAP) group. *Blue:* The early carotid atherosclerotic plaques (EAP) group. (B) Volcano plot of the differentially expressed genes (DEGs) in GSE28829. (C–E) The differences in the expressions of the 11 types of CTS between the AAP and EAP groups. The results are mean ± SEM (*n* = 13–16). ***p < 0.001, **p < 0.01, *p < 0.05, NS, indicates no significance; AAP *vs*. EAP group by Student’s *t*-test.

We removed the probe corresponding to multiple molecules. When the probe corresponding to the same molecule is encountered, only the probe with the largest signal value is retained (Jiang et al., 2020). We used the filtered data to check the standardization of the sample through the box chart (Fig. 2A), and we then used the ‘limma’ package to analyze the differences between two groups: the advanced atherosclerotic plaque (AAP) group and the early atherosclerotic plaque (EAP) group (Chen et al., 2021). Finally, we used the "limma" R package to conduct CSE28829 differentially expressed gene (DEG) screening at | log2 (fold-change)| >1, and we set adjusted *p*-values <0.05 as the thresholds of the DEG screening. These genes were visualized by ggplot2 ver. 3.3.3.

### Gene assays of cathepsin family member expressions

Eleven types of human CTS genes (CTSS, -C, -B, -Z, -H, -L, -K, -V,-W, -O, and -F) were screened as human CTS family members from the CSE28829 DEGs table. We calculated the DEG expressions of the CTS family members in the AAP and EAP groups by using this table and visualization.

### Integration of the PPI network and module analysis

STRING (ver. 11.0) was used to evaluate the interactive protein-protein interaction (PPI) relationships between the CSE28829 DEGs. We obtained the CTS-target gene set by selecting the CTS-target genes with a comprehensive score >0.4 after de-duplicating the genes.

### CTS-target gene set annotation

All CTS-target genes were converted into UniProt IDs and then mapped onto the Kyoto Encyclopedia of Genes and Genomes (KEGG) database. The KEGG online service tool ‘KEGG mapper’ was used to map the annotation results on the KEGG pathway database. We chose the best-matched KEGG pathway to display.

### Gene ontology (GO) and the pathway analysis of the CTS-target genes set

We visualized the CTS-target gene set by using the ggplot2 package and the Cluster Profiler R package. The threshold was set at *p* < 0.05.

### ssGSEA analysis

The scores of atherosclerotic plaques and normal plaques in GSE28829 were achieved by the ‘ssGSEA’ method in R package ‘GSVA,’ and the results are presented by heatmap mapping with the Pheatmap R package.

### Animal experiment

The animal study protocol (no. 30123) was conducted by Xianwu Cheng and approved by the institutional Animal Care and Use Committee of Nagoya University. CatS^−/−^mice (*n* = 10) were donated by Professor Shi Guoping of Harvard University and raised in Hall 1 of the Animal Experimental Facility/Clinical Research Building of Nagoya University. Wildtype mice (C57BL/6J, *n* = 18) were purchased and raised in the geriatrics biology room (605-2).

Mice in this experiment were 8 weeks old and weighed 21–25 g. The sample size is based on our previous study and ensured there are >5 animals in each group with study protocol number no. 30123 (Wang et al., 2019). In order to ensure the randomness of the experiment, we randomly selected mice (age and weight suitable for the experiment) in the breeding room. One week before the surgery, CatS^−/−^mice and wildtype mice were raised in the geriatrics biology room, All mice were maintained in a 22 °C room with a 12-h light/dark cycle and received standard chow and drinking water *ad libitum*.

We used 8-week-old male CTSS-deficient mice (CatS^−/−^ group) and wildtype (C57BL/6J) mice (WT; CatS^+/+^ group) for this experiment. Because intimal hyperplasia is pathologically similar to atherosclerotic disease, we used a carotid-artery ligation model as analogous to atherosclerosis. We conducted the carotid-artery ligation model as described (Wang et al., 2019). In brief, the right common carotid artery was dissected and ligated just proximal to its bifurcation with 5-0 silk ligature. All of the mice had survived as of 14 days post-surgery. On the sampling day, all mice were sacrificed with an intraperitoneal injection with an overdose of sodium pentobarbital (50 mg/kg; Dainippon Pharmaceutical, Osaka, Japan). Blood was collected for a routine examination and then perfused with phosphate-buffered saline (PBS) under physiological pressure. Both carotid arteries were isolated for a pathological evaluation. Cross-cryosections (5-μm-thick) at 2 mm proximal to the ligated site were prepared and then stained with CD68 stain.

### Cell line and cell culture

The RAW 264.7 mouse macrophage cell line was obtained from the China Cell Line Bank (Beijing, China) and then cultured in Dulbecco’s modified Eagle’s medium (DMEM; Biological Industries, Kibbutz Beit Haemek, Israel; Lot: 2136252) supplemented with 10% heat-inactivated fetal bovine serum (FBS; Biological Industries, Kibbutz Beit Haemek, Israel; Lot: 2134232). After allowing adherence for 8 h, These cells were collected for two group: Lipopolysaccharides (LPS; Solarbio, Beijing, China; L8880) treatment (LPS (+) group; 1 μg ml^−1^) or not (LPS (−) group) (Du et al., 2017). After 24 h, these cells were collected and performed the corresponding RNA test.

### Gene expression assay

Total RNA was extracted from the RAW 264.7 mouse macrophage cell with the use of an RNeasy Fibrous Tissue Mini-Kit (Qiagen, Hilden, Germany) and subjected to reverse transcription (Wang et al., 2019). The resulting cDNA was subjected to a quantitative real-time polymerase reaction chain (RT-PCR) analysis with primers specific for CTSS, p22^phox^, p47^phox^, gp91^phox^ with the use of an 7300 Plus Real-Time PCR System (Applied Biosystems, Foster City, CA, USA). The transcription of targeted RNAs was normalized to glyceraldehyde 3-phosphate dehydrogenase (GAPDH) mRNA levels. The primer sequences are listed in Table 4.

### Statistical analyses

Data are expressed as the mean ± standard deviation (SD). We performed comparisons of two groups by using Student’s *t*-test with SPSS software ver. 19.0 (SPSS, Chicago, IL, USA) or R ver. 3.6.3. Based on the Kolmogorov–Smirnov normality test, the data showing a skewed distribution and the expression profiles of genes of interest or predefined gene sets between clusters were compared using the Mann–Whitney *U*-test. A *p*-value <0.05 was considered significant. All morphological analyses were evaluated by two observers in a blind manner, and the values they obtained were averaged by two observers.

## Results

### Identification of DEGs associated with atherosclerosis

#### The DEGs gene selected from atherosclerosis

The EAP group was comprised of 13 samples of early atherosclerotic plaque from the GSE28829 dataset, and the AAP group was 16 samples of advanced atherosclerotic plaque provided by GSE28829. With the limma analysis, the expression of a total of 21,655 genes was identified in atherosclerotic lesions, and among them a total of 275 GSE28829-DEGs were identified at thresholds of | log2 (fold-change)| >1 and adjusted *p* < 0.05. These genes include 232 upregulated genes and 43 downregulated genes (Fig. 2B).

#### CTSS is the most DEG in cathepsin family in progress of atherosclerosis

We selected 11 human CTS family genes from the atherosclerotic gene expression (Table 1). Only three members of the human CTS family (CTSS, CTSC, and CTSB) showed significant differences (log2 (fold-change) >1) between the AAP and EAP groups. According to the data, we know that CTSS is the most differentially expressed gene (log2 (fold-change) = 1.78) in atherosclerosis. The differences in the expressions of CTS genes between the two groups are represented by the violin diagram (Figs. 2C–2E).

**Table 1:** Differences in gene expressions of cathepsin family members in the GSE28829 dataset. CTSS, CTSC, and CTSB are the main target molecules in the CTS family that are involved in atherosclerosis.

Gene symbol	logFC	AveExpr	*P* Value	adj. *P* Value
CTSS	1.783707971	8.198067212	1.83E−07	6.40E−05
CTSC	1.270319774	8.307986856	7.14E−07	0.000148572
CTSB	1.118294678	11.21555121	3.87E−08	2.95E−05
CTSZ	0.960217829	7.577926592	5.34E−05	0.00210386
CTSH	0.768094423	9.485990238	0.000653495	0.012074603
CTSL	0.767289644	9.294853877	3.48E−07	9.54E−05
CTSK	0.563023244	10.09637165	0.003741624	0.040983752
CTSV	0.245554858	5.662721577	0.00571263	0.055127895
CTSW	0.186911222	5.579753232	0.108736528	0.383811537
CTSO	0.028581154	8.538800486	0.813836025	0.941628038
CTSF	0.015970996	8.076471611	0.902332601	0.969789867

### PPI network analysis

The PPI network of 275 GSE28829-DEGs was constructed using the STRING database, and we selected CTS-target genes with a comprehensive score >0.4 after de-duplicating the genes. The result was a set of 61 CTS-target genes.

### Gene ontology and pathway enrichment analysis

We conducted a gene ontology (GO) and KEGG pathway analysis using the 61 CTS-target genes. Under the condition of *p* adj. < 0.05, there are 575 biological process (BP), 76 cellular component (CC), 36 molecular function (MF), and 46 KEGG pathways. We selected the top five results of the page to present, and the top three results for visualization.

#### CTSS related gene is closely related to phagocytosis and phagosome related cells

The results revealed that the genes that had significant roles in the formation of a BP were neutrophil activation (GO:0042119), neutrophil degranulation (GO:0043312), neutrophil activation involved in an immune response (GO:0002283), neutrophil-mediated immunity (GO:0002446), and phagocytosis (GO:0006909).

#### The main genes of CTSS are distributed in the phagocyte-related structures

The top five CC genes were endocytic vesicle (GO:0030139), tertiary granule (GO:0070820), endocytic vesicle membrane (GO:0030666), secretory granule membrane (GO:0030667), and MHC class II protein complex (GO:0042613). The top five MF genes were amyloid-beta binding (GO:0001540), peptide binding (GO:0042277), amide binding (GO:0033218), signaling pattern recognition receptor activity (GO:0008329), and pattern recognition receptor activity (GO:0038187).

#### CTSS related genes were mainly enriched in phagosome pathway

**Figure 3:**
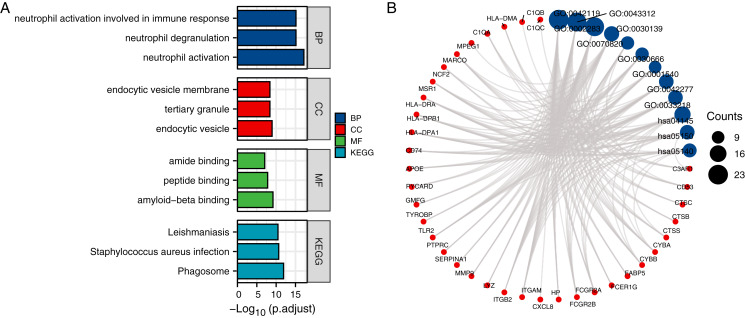
Identification of CTS-related genes and functional analysis results. (A) Bubble diagram of the KEGG pathway and GO enrichment and KEGG analyses of CTS-related genes; the top three information of each part is used for drawing and display. (B) The relationship between the top three genes in each part of GO enrichment and KEGG pathway analysis, terms in dot plot were ordered by count number. BP, biological process; GO, gene ontology; KEGG, Kyoto Encyclopedia of Genes and Genomes.

**Figure 4:**
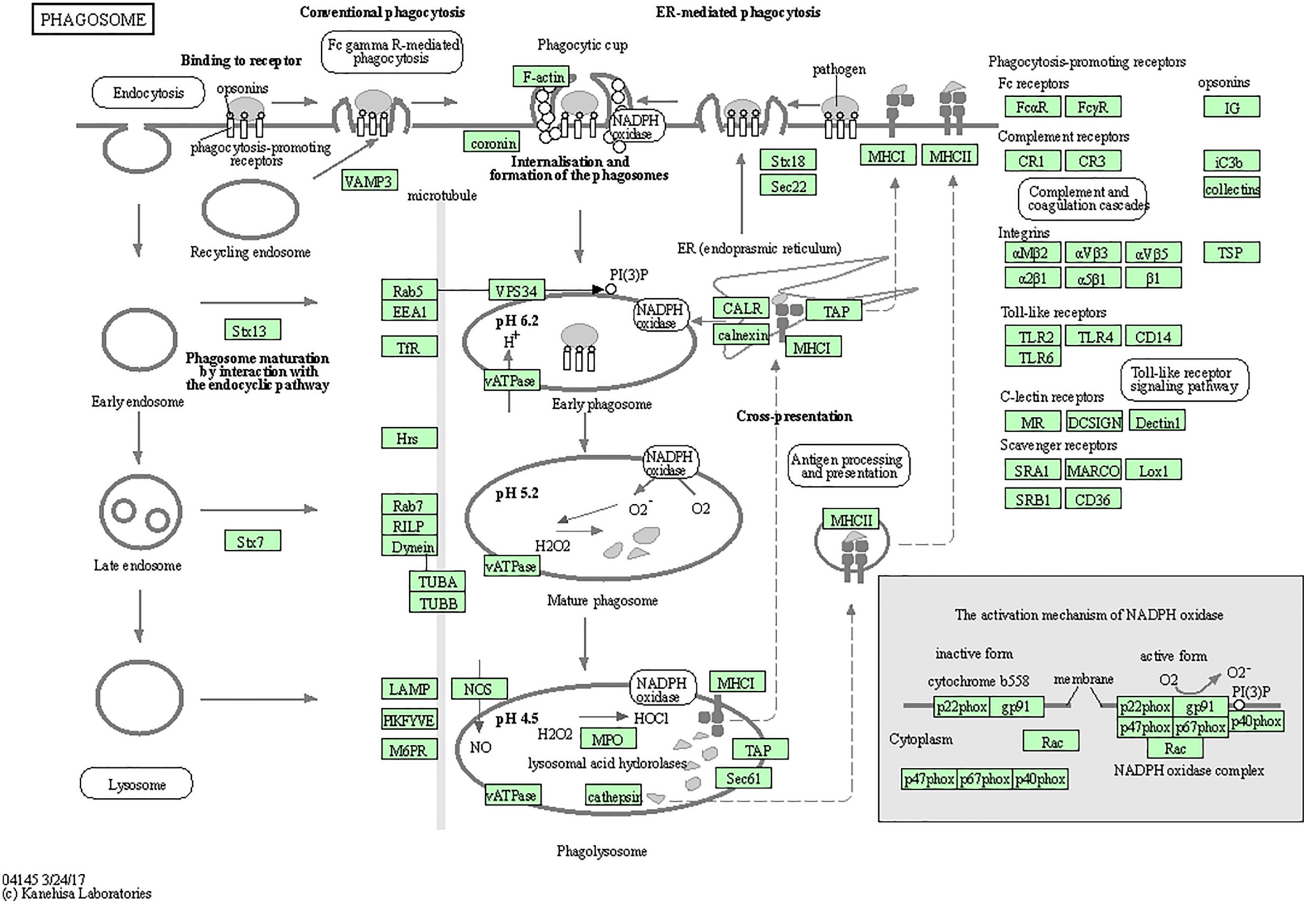
Cathepsin family target genes in phagosomes. Fifteen genes (CTSS, CYBA, CYBB, FCGR2A, FCGR2B, HLA-DMA, HLA-DPA1, HLA-DPB1, HLA-DRA, ITGAM, ITGB2, MSR1, NCF2, TLR2, and MARCO) were significantly enriched in the phagosome signaling pathway.

The top five enriched KEGG pathways were KEGG-Phagosome (hsa04145), KEGG-*Staphylococcus aureus* infection (hsa05150), KEGG-Leishmaniasis (hsa05140), KEGG-Tuberculosis (hsa05152), and KEGG-Pertussis (hsa05133) (Table 2). The top five KEGG pathway target genes had been selected from the GO analysis table (Table 3), and these data were then visualized (Figs. 3 and 4).

**Table 2:** Go and KEGG analysis of the five top CTS-related genes.

Ontology	ID	Description	GeneRatio	*p* adjust	*q* Value
BP	GO:0042119	Neutrophil activation	23/60	6.83E−18	4.09E−18
BP	GO:0043312	Neutrophil degranulation	21/60	7.49E−16	4.48E−16
BP	GO:0002283	Neutrophil activation involved in immune response	21/60	7.49E−16	4.48E−16
BP	GO:0002446	Neutrophil mediated immunity	21/60	8.86E−16	5.3E−16
BP	GO:0006909	Phagocytosis	17/60	5.07E−13	3.03E−13
CC	GO:0030139	Endocytic vesicle	13/61	1.11E−09	5.08E−10
CC	GO:0070820	Tertiary granule	10/61	4.05E−09	1.86E-09
CC	GO:0030666	Endocytic vesicle membrane	10/61	4.05E−09	1.86E−09
CC	GO:0030667	Secretory granule membrane	12/61	4.05E−09	1.86E−09
CC	GO:0042613	MHC class II protein complex	5/61	3.22E−08	1.48E−08
MF	GO:0001540	Amyloid-beta binding	9/58	6.6E−10	4.7E−10
MF	GO:0042277	Peptide binding	12/58	1.57E−08	1.12E−08
MF	GO:0033218	Amide binding	12/58	8.93E−08	6.37E−08
MF	GO:0008329	Signaling pattern recognition receptor activity	4/58	0.0000225	0.0000161
MF	GO:0038187	Pattern recognition receptor activity	4/58	2.25E−05	0.0000161
KEGG	hsa04145	Phagosome	15/51	1.19E−12	6.15E−13
KEGG	hsa05150	*Staphylococcus aureus* infection	12/51	2.18E−11	1.12E−11
KEGG	hsa05140	Leishmaniasis	11/51	3.37E−11	1.74E−11
KEGG	hsa05152	Tuberculosis	13/51	1.26E−09	6.5E−10
KEGG	hsa05133	Pertussis	9/51	1.61E−08	8.3E−09

**Note:**

BP: biological process, GO: gene ontology, KEGG: Kyoto Encyclopedia of Genes and Genomes.

**Table 3:** Expression of CTS-related genes in the top five signaling pathways in the KEGG enrichment analysis. These molecules participate in the progression of atherosclerosis mainly by mediating the phagosome.

Ontology	Description	*p* adjust	geneID	Count
KEGG	Phagosome	1.19178E−12	CTSS/CYBA/CYBB/FCGR2A/FCGR2B/HLA-DMA/HLA-DPA1/HLA-DPB1/HLA-DRA/ITGAM/ITGB2/MSR1/NCF2/TLR2/MARCO	15
KEGG	*Staphylococcus aureus* infection	2.17596E−11	C1QA/C1QB/C1QC/C3AR1/FCGR2A/FCGR2B/HLA-DMA/HLA-DPA1/HLA-DPB1/HLA-DRA/ITGAM/ITGB2	12
KEGG	Leishmaniasis	3.37445E−11	CYBA/CYBB/FCGR2A/HLA-DMA/HLA-DPA1/HLA-DPB1/HLA-DRA/ITGAM/ITGB2/NCF2/TLR2	11
KEGG	Tuberculosis	1.25981E−09	CD74/CTSS/FCER1G/FCGR2A/FCGR2B/HLA-DMA/HLA-DPA1/HLA-DPB1/HLA-DRA/IL10RA/ITGAM/ITGB2/TLR2	13
KEGG	Pertussis	1.60888E−08	C1QA/C1QB/C1QC/IRF8/CXCL8/ITGAM/ITGB2/LY96/PYCARD	9

**Table 4:** Primer sequences used for quantitative real-time PCR

Genes	Forward Primers	Reverse Primers
p22^phox^	AACTACCTGGAGCCAGTTGAG	AATTAGGAGGTGGTGGAATATCGG
gp91^phox^	ACTTTCCATAAGATGGTAGCTTGG	GCATTCACACACCACTCAACG
p47^phox^	CTGAGGGTGAAGCCATTGAGG	GCCGGTGATATCCCCTTTCC
CatS	GTGGCCACTAAAGGGCCTG	ACCGCTTTTGTAGAAGAAGAAGGAG
GAPDH	ATGTGTCCGTCGTGGATCTGA	ATGCCTGCTTCACCACCTTCT

**Note:**

CatS, cathepsin S; GAPDH, gluceradehyde-3-phosphate dehydrogenase.

### Animal experiment analysis

**Figure 5:**
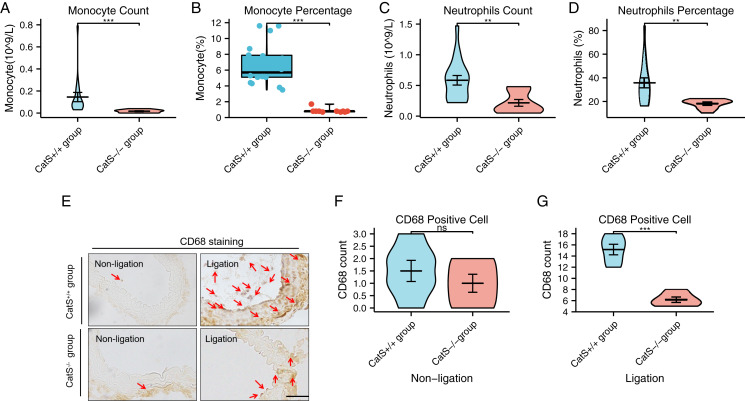
Identification of target cells for phagosomes. (A and B) The blood Monocyte count and percentage for the CatS^+/+^ group and CatS^−/−^ group. (C and D) The blood neutrophils count and percentage display for the two groups. (E–G) Representative picture and statistical results of macrophage (CD68 staining) infiltration in the carotid artery injury model. Results are mean ± SD (*n* = 6–18). Scale bar: 100 μm. ***p < 0.001, **p < 0.01, NS indicates no significance. CatS^+/+^ group *vs*. CatS^−/−^ group by Student’s *t*-test or Mann–Whitney *U* test.

Wildtype C57BL/6J mice and CatS^−/−^ mice were anesthetized *via* the abdominal cavity 14 days after the ligation of the carotid artery; blood samples were collected by apical puncture, and a routing blood analysis was performed. The blood testing showed that the CatS^−/−^ mice had a significantly lower monocyte count (0.02 ± 0.01 *vs*. 0.15 ± 0.18, *p* < 0.001), lower monocyte percentage (0.86 ± 0.30% *vs*. 6.73 ± 2.61%, *p* < 0.001), lower neutrophil count (0.22 ± 0.18 *vs*. 0.58 ± 0.33, *p* = 0.001), and lower neutrophil percentage (18.04 ± 3.96% *vs*. 35.74 ± 19.00%, *p* = 0.005) than the CatS^+/+^ group (Figs. 5A–5D). CD68 staining revealed that in the ligated but not in the non-ligated carotid arteries, the presence of CTSS significantly increased the macrophage infiltration (6.17 ± 1.17 *vs*. 15.17 ± 2.32, *p* < 0.001) (see Figs. 5E–5G). These results suggest that CTSS participates in phagosomes, mainly through neutrophils, monocytes, and macrophages.

### ssGSEA analysis

**Figure 6:**
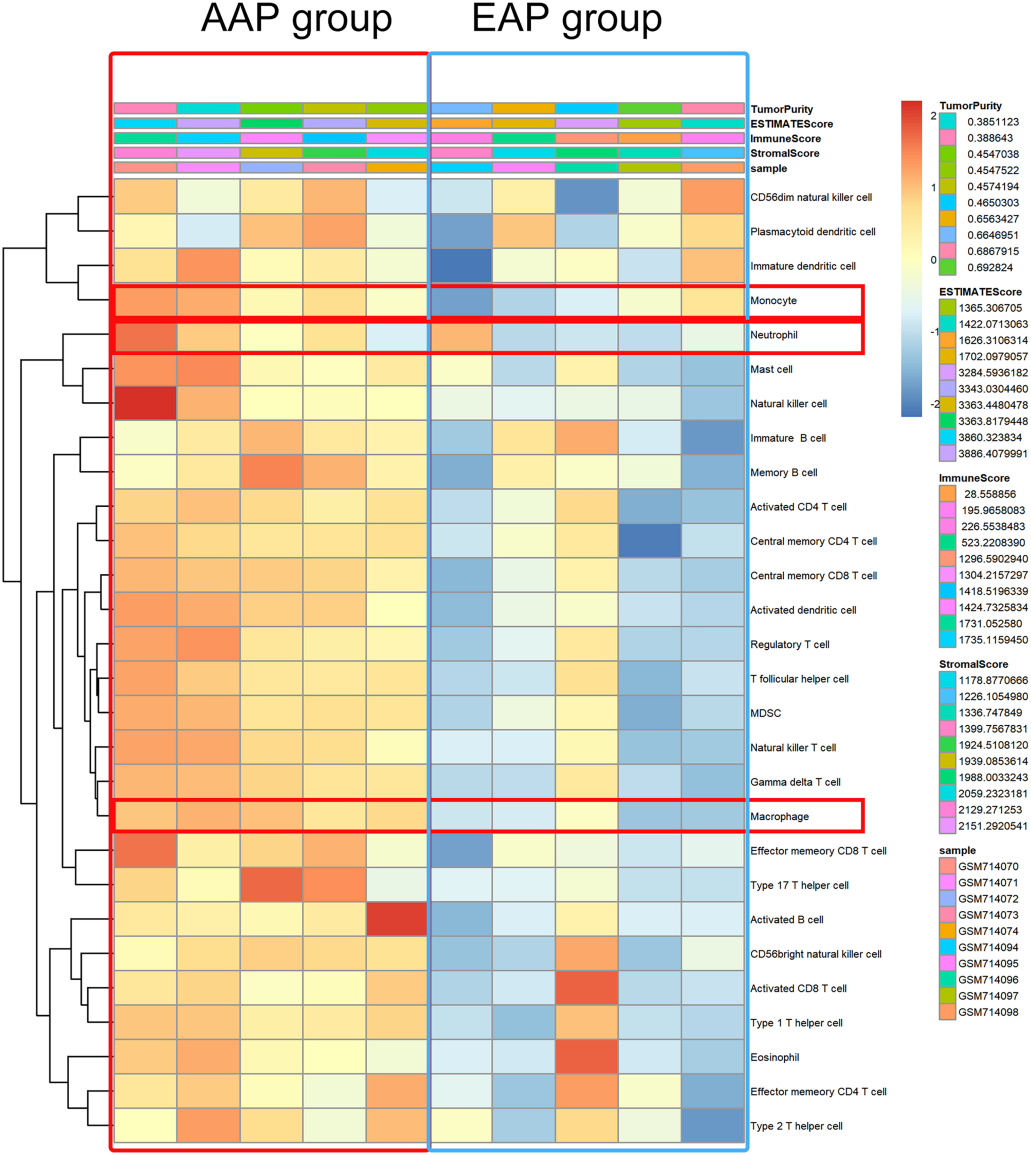
Phagosome-related cells were enriched in atherosclerotic plaque. Phagosome-related cells (macrophage, monocytes, and neutrophils) were enriched in the AAP group.

Since each sample gene was extracted from GSE28829 database, we speculated that the number of phagosome-related cells in the AAP group was higher than that in the EAP group. We thus composed a heatmap with ssGSEA to visualize the relative abundance of 28 immune infiltrating cell subpopulations from the selected dataset (Fig. 6). Phagosome-related cells (macrophage, monocytes, and neutrophils) were enriched in the AAP group.

### Macrophage play an important role in phagosome

**Figure 7:**
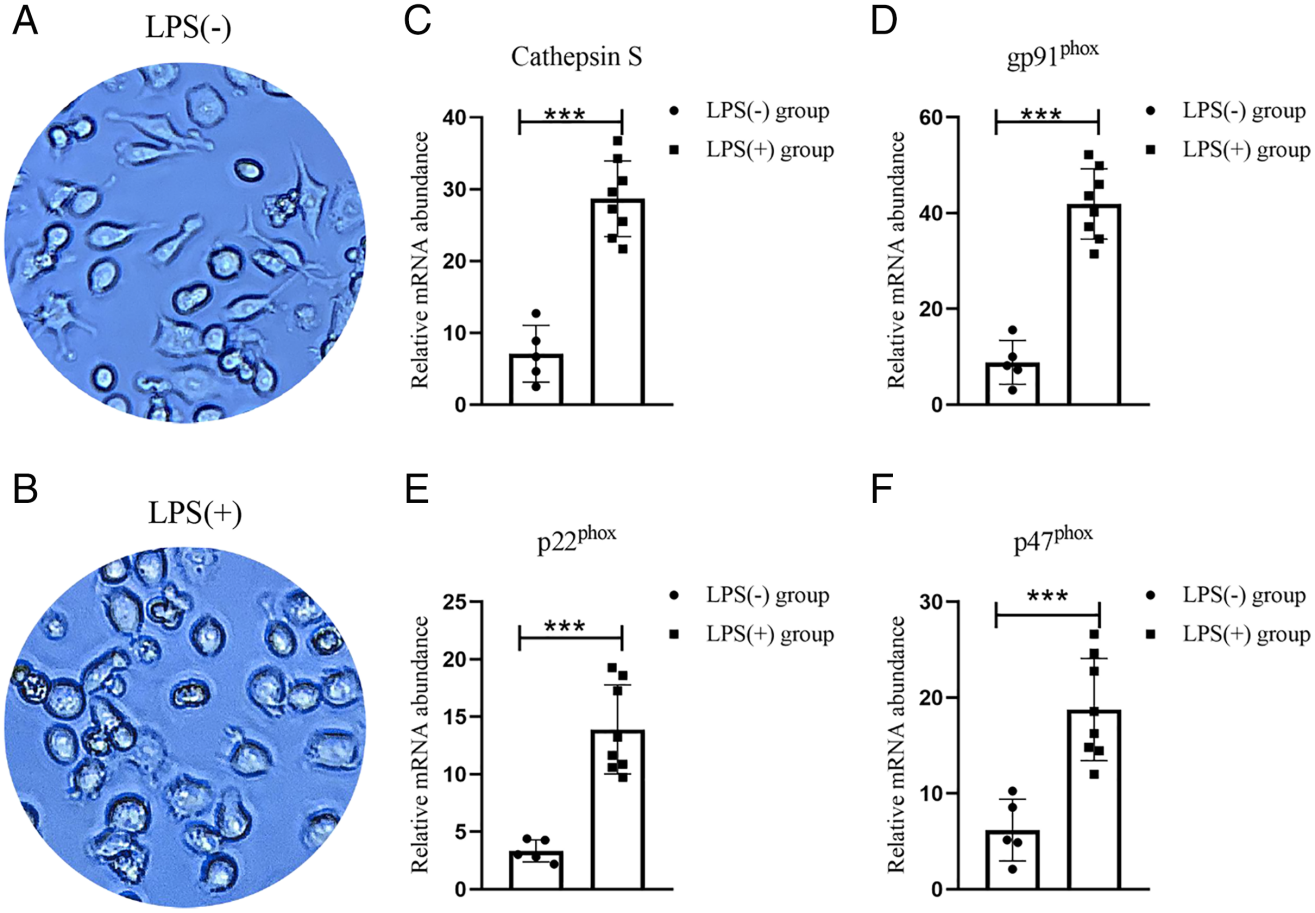
Identify the phagosome marker genes of macrophage. (A and B) The corresponding representative picture without/with LPS-stimulated RAW264.7 cells; (C–F) The expression of CTSS, and phagosome marker genes (gp91^phox^, p22^phox^, p47^phox^) expression in LPS-stimulated macrophages. ***p < 0.001 vs. LPS (-) group by Student’s *t*-test.

Both animal study and ssGSEA analysis had showed that macrophage as an important phagosome cell. To assess whether macrophage expression the phagosome target molecular *in vitro*, we use LPS to stimulate RAW 264.7 mouse macrophage cell (Figs. 7A and 7B). Real-time PCR analysis of the mRNA expressions of gp9^1phox^, p22^phox^, p47^phox^ demonstrate increased expression of these phagosome marker genes in macrophage (Figs. 7D and 7E) (Canton, 2014). What is more, The expression trend of CTSS was consistent with these Phagosome marker genes (Fig. 7C).

## Discussion

Atherosclerosis is the prototypical chronic inflammatory disease associated with an activated innate immune response (Liu et al., 2018). According to the current research, members of the CTS family have the function of communicating inflammation and immunity (Liu et al., 2018; Wang et al., 2019), and it is thus of great value to understand CTS-related cells and associated biological functions in order to further improve the treatment of atherosclerotic diseases. We hypothesized that CTS family members (CTSS, CTSB, and CTSC— especially CTSS) might be appropriate for assessing the phagosome pathway of advanced carotid plaques with a GSE28829 gene analysis, and we would then verify the analysis results by blood testing and immunohistochemical staining in CatS^−/−^mice and by an ssGSEA analysis. We speculated that the results could indicate whether CTS family members mediate the participation of neutrophils, macrophages, and monocytes in phagosomes.

Animal model studies have shown that many CTS family genes are highly expressed in atherosclerotic plaques, but there is a lack of unified research on the total expression of CTS family genes in atherosclerotic plaques (Kitamoto et al., 2007; Wang et al., 2019). This is also true of the sporadic clinical sample experiments (Stellos et al., 2016; Bibli et al., 2019). High-throughput sequencing can clarify the expression of human atherosclerosis-related genes and provide a theoretical reference for screening GSE28829 dataset CTS-target genes. Eleven types of CTS family genes have been identified in humans (Cheng et al., 2011). We selected the CTS-target genes from a gene expression table. Based on the IogFc values, we observed that the differential expressions of CTSS, CTSB, and CTSC were the highest in atherosclerotic plaques and the expressions were positively expressed in advanced plaques, indicating a positive trend of promoting atherosclerosis. In Figs. 2C–2E, it can be seen that CTSS, CTSB, and CTSC genes show significance differences between the EAP and AAP groups. These results are consistent with reports that CTSS, CTSB, and CTSC promoted atherosclerosis (Sukhova et al., 2003; Kitamoto et al., 2007; Liu et al., 2017; Wang et al., 2019).

To identify the GSE28829-DEGs, we screened the GSE28829 dataset at thresholds of |log2 (fold-change)| >1 and adjusted *p* < 0.05, revealing 275 GSE28829-DEGs. To identify the GSE28829-DEGs involved in the progression of atherosclerotic plaques, we constructed a GSE28829-DEGs PPI network and selected the 61 CTS-target genes by using the STRING online database.

The GO and KEGG pathway enrichment analysis revealed that these genes were significantly enriched in phagosome pathways. Many types of cells have been shown to be involved in the phagosome process (*e.g*., neutrophils, dendritic cells (DCs), monocytes, and macrophages), and many other cell types (*e.g*., epithelial cells, fibroblasts, and some B-lymphocyte subsets) are also involved in phagocytosis (Steigbigel, Lambert & Remington, 1974; Goldman, 1977; Li et al., 2006; Sartor & Muehlbauer, 2007; Levin, Grinstein & Canton, 2016; Pauwels et al., 2017). Indeed, our present study’s CC results revealed that these genes are enriched in endocytic vesicles and that the MF genes for CTS are involved mainly in peptide binding and amide binding. These results are supported by a report that CTSS is the main processing enzyme in spleen cells and DCs, which affects the entry of antigens into the immune system (Shi et al., 1999).

In addition, in the present GO analysis, the MF genes involved in neutropenia and neutropenia activation are also involved in the immune response. At the same time, a related KEGG pathway also involves the phagosome. These data were supported by the results of the present animal experiment, as the number and percentage of neutrophils in blood in the CatS^−/−^ group also decreased correspondingly. A 2014 study indicated that there is no M1 or M2 polarization of macrophages in atherosclerosis, and that only an M1 gene set was increased in foam cells from atherosclerotic subjects compared to foam cells from healthy subjects (da Rocha, De Bastiani & Klamt, 2014). That study showed that although the expression of macrophages is increased in atherosclerotic diseases, the polarization of macrophages is not obvious.

According to a description from Novak & Koh (2013), M1 macrophages are classically activated macrophages, also known as “killer” macrophages, and M2 macrophages are alternately activated macrophages, also known as “therapeutic” macrophages. As the most important aspect of macrophages, both M1 and M2 macrophages express CD68 (Tang, Nikolic-Paterson & Lan, 2019). Herein, we used CD68 to represent macrophages in a broad sense (Iglesias-Linares & Hartsfield, 2017). Our immunohistochemical results revealed that CTSS deletion reduced the infiltration of CD68 cells compared to the wildtype mice. Our findings also help clarify the pathway for CTS-target genes in atherosclerosis. We used CatS^−/−^ mice to clarify the phagosome process in a carotid ligation model. In their article on cerebrovascular accident, Carmona-Mora et al. (2021) pointed out that monocytes and neutrophils are the main types of reactive cells. As the most important phagosome-related cells in the human body, neutrophils and monocytes may be closely related to vascular injury and repair. It has also been reported that macrophages play a role in atherosclerosis through the endocytosis of lipoprotein deposits and the phagocytosis of apoptotic cells (Yin et al., 2020). At the same time, myeloperoxidase (MPO) from neutrophils and macrophages has been confirmed to be a type of immune enzyme, and its drug inhibitor has a positive protective effect on arteriosclerotic heart disease (Soubhye, Van Antwerpen & Dufrasne, 2020). By performing an ssGSEA analysis, we observed that phagosome-related cells (macrophages, monocytes, and neutrophils) were enriched in the AAP group, consistent with our hypothesis.

Previous *in vivo* experiments have found that CTSS deficiency and its special drug inhibitors reduce the expression of gp91^phox^, p22^phox^, p47^phox^, and decrease the infiltration of macrophages (Wang et al., 2019). These experimental data prove that CTSS is closely related to phagosome-related cells: macrophages. In this study, we used GSE28829 to confirm that CTSS is the most DEG of human atherosclerotic cathepsin family. With the help of bioinformatics GO and KEGG pathway enrichment analysis, we found that CTSS-related genes are closely related to phagosome. In order to verify it, we used ssGSEA analysis, which further confirmed the aggregation of phagosome-related macrophages in arterial plaques. According to previous study that phagosome-associated molecules (gp91^phox^, p22^phox^, p47^phox^) are highly expressed in macrophages (Canton, 2014). We have carried out *in vitro* experiments and found that this phagosome marker genes are highly expressed in macrophage. Although we have not proved the relationship between CTSB, CTSC and neutrophils and monocytes, but the above experimental results, at least suggest that CTSS plays a role in atherosclerotic disease by participating in phagosome *via* macrophage.

Several study limitations should be considered. First, concerning the *in vivo* experiments, although the expression levels of the target genes were obtained by microarray, we did not obtain their protein expression levels. Second, Although CTSS were found to be closely related to phagosome *via* macrophage in animal and *in vitro* cell experiments, we do not evaluate CTSS role in other phagosome related cells.

## Conclusions

Scattered reports suggest that cathepsins are involved in the inflammation and immune response in atherosclerosis, but the expression of CTS family members in atherosclerosis and the signal pathways involved are not clear. Our present findings revealed that among the CTS family members, the expressions of CTSS, CTSB, and CTSC are the most significant in atherosclerotic progression and are the main CTSs involved in phagocytosis, which plays an important role in the immune response. In the process of atherosclerosis involving CTSs, CTSS are the main target molecules in the CTS family that are involved in atherosclerosis. These molecules participate in the progression of atherosclerosis by mediating the phagosome *via* macrophage. CTSS involved in the phagosome may provide a corresponding therapeutic target for the immunotherapy of atherosclerosis in the future.

## Supplemental Information

10.7717/peerj.12846/supp-1 Supplemental Information 1 ARRIVE 2.0 Checklist.

10.7717/peerj.12846/supp-2 Supplemental Information 2 Figure 5 raw data.

10.7717/peerj.12846/supp-3 Supplemental Information 3 Fig5. Raw data with format xlsx.

10.7717/peerj.12846/supp-4 Supplemental Information 4 Figure 7 raw data.

## Additional Information and Declarations

### Data Availability

The following information was supplied regarding data availability: The raw data is available in the Supplemental Files and at GEO: GSE28829.
