# Supplementary material for: Cathepsin S are involved in human carotid atherosclerotic disease progression, mainly by mediating phagosomes: bioinformatics and in vivo and vitro experiments
Source: PeerJ. 2022 Feb 8;10:e12846. doi: 10.7717/peerj.12846 (PMC8833225; doi:10.7717/peerj.12846)
Supplement: Supplemental Information 2 [file peerj-10-12846-s002.pdf]

# Fig.5 data

| G | MN | ML | Mc | Mp | Nc | Np |
|---|---|---|---|---|---|---|
| 1 | 0 | 16 | 0.79 | 11.6 | 1.47 | 83.9 |
| 1 | 1 | 13 | 0.29 | 8.7 | 1.01 | 62.2 |
| 1 | 2 | 12 | 0.22 | 11.6 | 0.98 | 54.7 |
| 1 | 3 | 17 | 0.2 | 5.3 | 0.72 | 49.6 |
| 1 | 2 | 18 | 0.19 | 7.9 | 0.69 | 42.1 |
| 1 | 1 | 15 | 0.12 | 11 | 0.68 | 41.5 |
| 1 | | | 0.11 | 7.5 | 0.65 | 37.3 |
| 1 | | | 0.1 | 5.2 | 0.64 | 34.8 |
| 1 | | | 0.09 | 3.8 | 0.61 | 33.9 |
| 1 | | | 0.09 | 7.8 | 0.59 | 29.9 |
| 1 | | | 0.08 | 5.1 | 0.56 | 29.7 |
| 1 | | | 0.07 | 4.3 | 0.35 | 29.1 |
| 1 | | | 0.06 | 3.5 | 0.31 | 26.9 |
| 1 | | | 0.05 | 4.4 | 0.28 | 19.3 |
| 1 | | | 0.04 | 6.1 | 0.26 | 18.7 |
| 1 | | | 0.04 | 5.1 | 0.26 | 17.3 |
| 1 | | | 0.04 | 7 | 0.24 | 16.3 |
| 1 | | | 0.03 | 5.2 | 0.22 | 16.2 |
| 2 | 1 | 5 | 0.04 | 0.8 | 0.05 | 10.2 |
| 2 | 0 | 6 | 0.03 | 1.7 | 0.07 | 13.8 |
| 2 | 2 | 7 | 0.03 | 0.8 | 0.09 | 14.6 |
| 2 | 1 | 8 | 0.02 | 0.8 | 0.09 | 18.4 |
| 2 | 0 | 5 | 0.02 | 0.7 | 0.1 | 18.5 |
| 2 | 2 | 6 | 0.02 | 0.8 | 0.17 | 19.9 |
| 2 | | | 0.01 | 0.7 | 0.21 | 19.9 |
| 2 | | | 0.001 | 0.8 | 0.43 | 20.4 |
| 2 | | | 0.002 | 0.7 | 0.47 | 22.3 |
| 2 | | | 0.003 | 0.8 | 0.48 | 22.4 |

G:group(1:CatS$^{+/+}$;2:CatS$^{-/-}$)
MN:CD68 Non-ligation
ML:CD68 Ligation
Mc:Monocyte Count
Mp:monocyte Percentage
Nc:Neutrophil Count
Np:Neutrophil Percentage

# Statistical description

| | G | N | mean | SD | SEM |
|---|---|---|---|---|---|
| MN | CatS+/+ | 6 | 1.5 | 1.0488088 | 0.4281744 |
| | CatS-/- | 6 | 1 | 0.8944272 | 0.3651484 |
| ML | CatS+/+ | 6 | 15.166667 | 2.3166067 | 0.9457507 |
| | CatS-/- | 6 | 6.166667 | 1.1690452 | 0.4772607 |
| Mc | CatS+/+ | 18 | 0.145 | 0.1765769 | 0.0416196 |
| | CatS-/- | 10 | 0.0176 | 0.0134015 | 0.0042379 |
| Mp | CatS+/+ | 18 | 6.727778 | 2.6067346 | 0.6144132 |
| | CatS-/- | 10 | 0.86 | 0.2988868 | 0.0945163 |
| Nc | CatS+/+ | 18 | 0.584444 | 0.3302445 | 0.0778394 |
| | CatS-/- | 10 | 0.216 | 0.1751951 | 0.0554016 |
| Np | CatS+/+ | 18 | 35.744444 | 17.9945162 | 4.2413482 |
| | CatS-/- | 10 | 18.04 | 3.9618178 | 1.2528368 |

# Normality detection and p-value acquisition

| Normality test | | | |
|---|---|---|---|
| Kolmogorov-Smirnov[a] | | | |
| | statistic | df | Sig. |
| MN | 0.198 | 12 | .200* |
| ML | 0.203 | 12 | 0.187 |
| Mc | 0.255 | 12 | 0.03 |
| Mp | 0.267 | 12 | 0.018 |
| Nc | 0.26 | 12 | 0.024 |
| Np | 0.25 | 12 | 0.036 |
| a. Lilliefors Significant level correction | | | |
| *. This is the lower limit of true significance. | | | |

**Student's t-test**

| | | F | Sig. | t | df | Sig.(both sides) |
|---|---|---|---|---|---|---|
| MN | Assuming the variance is equal | 0.313 | 0.588 | 0.889 | 10 | 0.395 |
| | Assuming the variance is not equal | | | 0.889 | 9.757 | 0.396 |
| ML | Assuming the variance is equal | 3.055 | 0.111 | 8.496 | 10 | 0 |
| | Assuming the variance is not equal | | | 8.496 | 7.391 | 0 |

**Mann-Whitney U test**

| | Mc | Mp | Nc | Np |
|---|---|---|---|---|
| Mann-Whitney U | 3.5 | 0 | 21 | 31 |
| Wilcoxon W | 58.5 | 55 | 76 | 86 |
| Z | -4.158 | -4.34 | -3.309 | -2.829 |
| Sig.(both sides) | 0 | 0 | 0.001 | 0.005 |
